# [(5-Bromo-1*H*-indol-3-yl)meth­yl]dimethyl­aza­nium nitrate

**DOI:** 10.1107/S1600536811020034

**Published:** 2011-06-18

**Authors:** Qing Wang, Zhong-Ye Fu, Xia Li, Liang-Min Yu

**Affiliations:** a Education Ministry Key Laboratory of Marine Chemistry and Technology, Ocean University of China, Qingdao, People’s Republic of China

## Abstract

In the title compound, C_11_H_14_BrN_2_^+^·NO_3_^−^, inter­molecular N—H⋯O and N—H⋯N hydrogen bonds link the proton­ated 5-bromo­gramine cation and the nitrate anions. Further N—H⋯O hydrogen bonds link the cation–anion pairs into a chain running parallel to [100]. C—H⋯O hydrogen bonds link the chains, forming a layer parallel to (001).

## Related literature

For background to gramine ramification, see: Kon-ya *et al.* (1994 ▶); Rie *et al.* (1996 ▶); Li *et al.* (2008 ▶, 2009 ▶). For a related structure, see: Golubev & Kondrashev (1984 ▶). 
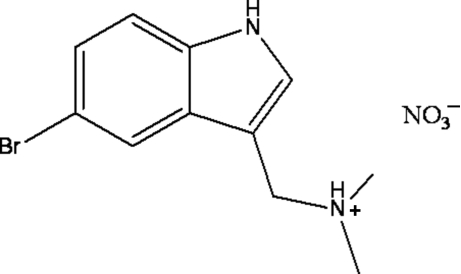


## Experimental

### 

#### Crystal data

C_11_H_14_BrN_2_^+^·NO_3_^−^
*M* *_r_* = 316.16
Orthorhombic, 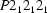

*a* = 9.1449 (2) Å
*b* = 10.8270 (3) Å
*c* = 13.1344 (3) Å
*V* = 1300.46 (5) Å^3^
*Z* = 4
Cu *K*α radiation
μ = 4.38 mm^−1^
*T* = 150 K
0.50 × 0.42 × 0.40 mm

#### Data collection

Agilent Gemini S Ultra CCD diffractometer
2543 measured reflections
1760 independent reflections
1713 reflections with *I* > 2σ(*I*)
*R*_int_ = 0.022
θ_max_ = 62.4°

#### Refinement

*R*[*F* ^2^ > 2σ(*F* ^2^)] = 0.034
*wR*(*F* ^2^) = 0.088
*S* = 1.07
1760 reflections
164 parameters
H-atom parameters constrained
Δρ_max_ = 0.60 e Å^−3^
Δρ_min_ = −0.77 e Å^−3^
Absolute structure: Flack (1983 ▶), 546 Friedel pairs
Flack parameter: −0.01 (3)

### 

Data collection: *CrysAlis PRO* (Agilent, 2010 ▶); cell refinement: *CrysAlis PRO*; data reduction: *CrysAlis PRO*; program(s) used to solve structure: *SHELXS97* (Sheldrick, 2008 ▶); program(s) used to refine structure: *SHELXL97* (Sheldrick, 2008 ▶); molecular graphics: *ORTEPIII* (Burnett & Johnson, 1996 ▶), *ORTEP-3 for Windows* (Farrugia, 1999 ▶) and *PLATON* (Spek, 2009 ▶); software used to prepare material for publication: *SHELXL97*.

## Supplementary Material

Crystal structure: contains datablock(s) I, global. DOI: 10.1107/S1600536811020034/dn2690sup1.cif

Structure factors: contains datablock(s) I. DOI: 10.1107/S1600536811020034/dn2690Isup3.hkl

Supplementary material file. DOI: 10.1107/S1600536811020034/dn2690Isup3.cml

Additional supplementary materials:  crystallographic information; 3D view; checkCIF report

## Figures and Tables

**Table 1:** Hydrogen-bond geometry (Å, °)

*D*—H⋯*A*	*D*—H	H⋯*A*	*D*⋯*A*	*D*—H⋯*A*
N1—H1⋯O2	0.91	2.24	3.041 (4)	146
N1—H1⋯O3	0.91	2.03	2.857 (4)	151
N1—H1⋯N3	0.91	2.49	3.391 (5)	169
N2—H2*D*⋯O2^i^	0.86	2.12	2.902 (4)	152
N2—H2*D*⋯O1^i^	0.86	2.65	3.388 (4)	144
C1—H1*B*⋯O3^ii^	0.96	2.45	3.293 (5)	146
C3—H3*B*⋯O3^ii^	0.97	2.40	3.259 (5)	147

Symmetry codes: (i) 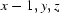; (ii) 
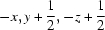
.

**Fig. 1.**
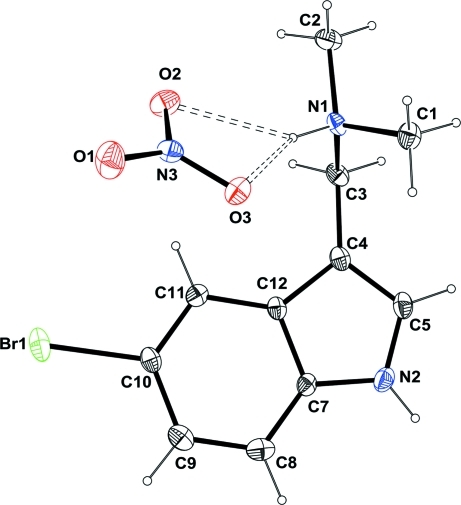
: A view of the molecule of (I), showing the atom-labelling scheme. Displacement ellipsoids are drawn at the 30% probability level. H atom are represented as small spheres of arbitrary radii. Hydrogen bonds are shown as dashed lines.

**Fig. 2.**
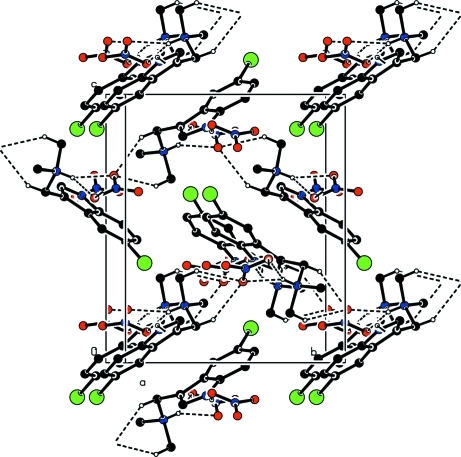
: Packing view of (I), showing the two-dimensional hydrogen-bonding layer. Hydrogen bonds are shown as dashed lines. H atoms not involved in hydrogen bondings have been omitted for clarity.

**Fig. 3.**
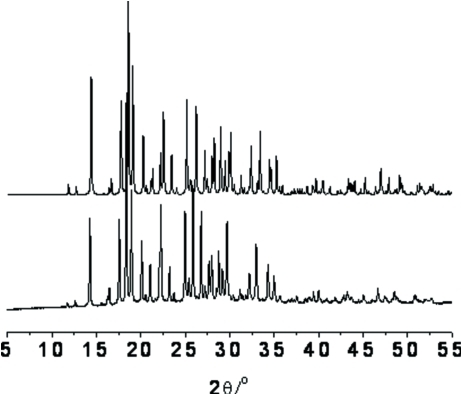
: The simulate X-ray powder diffraction pattern (upper) and the measured one (lower).

## supplementary crystallographic information

### Comment

Recently, gramine ramification was shown to be very efficient in preventing recruitment of larval settlement. Many compounds such as 2,5,6-Tribromo-1-methylgramine (Kon-ya *et al.*, 1994; Li *et al.*, 2008; Li *et al.* 2009) and 5,6-dichlorogramine (Rie *et al.*, 1996) have been reported. Here we report the synthesis and structure of the title compound (I).

The asymmetric unit contains one protonated 5-bromo-gramine and one NO_3_¯ anion linked by a bifurcated N—H···O hydrogen bonds (Table 1, Fig. 1). Futhermore, intermolecular N—H···O hydrogen bonds link the cation-anion couple to form a one-dimensional chain running parallel to the [100] direction (Table 1). These chains are further connected through C—H···O hydrogen bonds to form layer parallel to the (0 0 1) plane (Table 1, Fig. 2).

### Experimental

Eu(NO_3_)_3_.6H_2_O (0.2 mmol, 0.0892 g) was dissolved in CH_3_OH (5 ml), and then carefully layered onto a solution of 5-BrG (0.2 mmol, 0.0504 g) in C_2_H_5_OH (5 ml). After the solvent was evaporated to almost dry, pale-yellow block crystals suitable for X-ray analysis could be harvested.

For (I): C_11_H_14_BrN_3_O_3_ (316.15, %): calcd. C 41.97, H 2.716, N 12.95; found C 41.79, H 4.46, N 13.29.

X-ray powder diffraction pattern was recorded to check the solid-state phase purity of the bulky sample of compound (I). Supplementary Figure 3 shows the measured pattern and the simulated one on the basis of single-crystal analysis result.

### Refinement

All H atoms attached to C and N atoms were fixed geometrically and treated as riding with C—H = 0.93 Å (aromatic), 0.96 Å (methyl) or 0.97 Å (methylene) and N—H = 0.86 Å (amido) or 0.91Å (amonium) with *U*_iso_(H) = 1.2*U*_eq_(C_aromatic_, C_methylene_ or N) or *U*_iso_(H) = 1.5*U*_eq_(C_methyl_).

### Crystal data

**Table:**

C_11_H_14_BrN_2_^+^·NO_3_^−^	*F*(000) = 640
*M**_r_* = 316.16	*D*_x_ = 1.615 Mg m^−^^3^
Orthorhombic, *P*2_1_2_1_2_1_	Cu *K*α radiation, λ = 1.54178 Å
Hall symbol: P 2ac 2ab	Cell parameters from 2128 reflections
*a* = 9.1449 (2) Å	θ = 3.4–62.3°
*b* = 10.8270 (3) Å	µ = 4.38 mm^−^^1^
*c* = 13.1344 (3) Å	*T* = 150 K
*V* = 1300.46 (5) Å^3^	Block, yellow
*Z* = 4	0.50 × 0.42 × 0.40 mm

### Data collection

**Table:**

Agilent Gemini S Ultra CCD diffractometer	1713 reflections with *I* > 2σ(*I*)
Radiation source: fine-focus sealed tube	*R*_int_ = 0.022
graphite	θ_max_ = 62.4°, θ_min_ = 5.3°
Detector resolution: 16.0855 pixels mm^-1^	*h* = −10→10
φ and ω scans	*k* = −12→11
2543 measured reflections	*l* = −14→13
1760 independent reflections	

### Refinement

**Table:**

Refinement on *F*^2^	Hydrogen site location: inferred from neighbouring sites
Least-squares matrix: full	H-atom parameters constrained
*R*[*F*^2^ > 2σ(*F*^2^)] = 0.034	*w* = 1/[σ^2^(*F*_o_^2^) + (0.0583*P*)^2^] where *P* = (*F*_o_^2^ + 2*F*_c_^2^)/3
*wR*(*F*^2^) = 0.088	(Δ/σ)_max_ = 0.004
*S* = 1.07	Δρ_max_ = 0.60 e Å^−^^3^
1760 reflections	Δρ_min_ = −0.77 e Å^−^^3^
164 parameters	Extinction correction: *SHELXL97* (Sheldrick, 2008), Fc^*^=kFc[1+0.001xFc^2^λ^3^/sin(2θ)]^-1/4^
0 restraints	Extinction coefficient: 0.0131 (7)
Primary atom site location: structure-invariant direct methods	Absolute structure: Flack (1983), 546 Friedel pairs
Secondary atom site location: difference Fourier map	Flack parameter: −0.01 (3)

### Special details

**Table:**

Geometry. All e.s.d.'s (except the e.s.d. in the dihedral angle between two l.s. planes) are estimated using the full covariance matrix. The cell e.s.d.'s are taken into account individually in the estimation of e.s.d.'s in distances, angles and torsion angles; correlations between e.s.d.'s in cell parameters are only used when they are defined by crystal symmetry. An approximate (isotropic) treatment of cell e.s.d.'s is used for estimating e.s.d.'s involving l.s. planes.
Refinement. Refinement of *F*^2^ against ALL reflections. The weighted *R*-factor *wR* and goodness of fit *S* are based on *F*^2^, conventional *R*-factors *R* are based on *F*, with *F* set to zero for negative *F*^2^. The threshold expression of *F*^2^ > σ(*F*^2^) is used only for calculating *R*-factors(gt) *etc*. and is not relevant to the choice of reflections for refinement. *R*-factors based on *F*^2^ are statistically about twice as large as those based on *F*, and *R*- factors based on ALL data will be even larger.

### Fractional atomic coordinates and isotropic or equivalent isotropic displacement parameters (Å^2^)

**Table:**

	*x*	*y*	*z*	*U*_iso_*/*U*_eq_	
Br1	0.08934 (5)	0.37702 (4)	0.62866 (4)	0.0393 (2)	
N1	0.0708 (3)	0.7763 (3)	0.2870 (2)	0.0218 (7)	
H1	0.1132	0.7060	0.3108	0.026*	
N2	−0.3723 (3)	0.6214 (3)	0.3766 (2)	0.0293 (8)	
H2D	−0.4617	0.6044	0.3618	0.035*	
C1	0.0076 (5)	0.7478 (4)	0.1850 (3)	0.0286 (9)	
H1A	0.0844	0.7227	0.1396	0.043*	
H1B	−0.0396	0.8201	0.1584	0.043*	
H1C	−0.0625	0.6823	0.1914	0.043*	
C2	0.1875 (4)	0.8720 (4)	0.2783 (3)	0.0308 (9)	
H2A	0.2598	0.8452	0.2302	0.046*	
H2B	0.2325	0.8840	0.3436	0.046*	
H2C	0.1451	0.9483	0.2557	0.046*	
C3	−0.0444 (4)	0.8157 (4)	0.3639 (3)	0.0254 (8)	
H3A	0.0033	0.8342	0.4281	0.030*	
H3B	−0.0909	0.8909	0.3401	0.030*	
C4	−0.1584 (4)	0.7205 (4)	0.3813 (3)	0.0238 (8)	
C5	−0.2987 (4)	0.7213 (4)	0.3437 (3)	0.0285 (9)	
H5A	−0.3371	0.7822	0.3015	0.034*	
C7	−0.2829 (4)	0.5507 (4)	0.4374 (3)	0.0218 (8)	
C8	−0.3083 (5)	0.4384 (4)	0.4854 (3)	0.0284 (10)	
H8A	−0.3982	0.3989	0.4796	0.034*	
C9	−0.1965 (5)	0.3868 (4)	0.5421 (3)	0.0282 (9)	
H9A	−0.2104	0.3119	0.5753	0.034*	
C10	−0.0612 (4)	0.4490 (4)	0.5491 (3)	0.0245 (9)	
C11	−0.0335 (4)	0.5599 (4)	0.5012 (3)	0.0225 (9)	
H11A	0.0564	0.5992	0.5075	0.027*	
C12	−0.1466 (4)	0.6110 (4)	0.4426 (3)	0.0200 (8)	
N3	0.2674 (4)	0.5222 (3)	0.3505 (2)	0.0261 (8)	
O1	0.3385 (4)	0.4266 (3)	0.3625 (3)	0.0462 (8)	
O2	0.3106 (3)	0.6231 (3)	0.3859 (2)	0.0337 (7)	
O3	0.1487 (3)	0.5214 (3)	0.3021 (2)	0.0309 (7)	

### Atomic displacement parameters (Å^2^)

**Table:**

	*U*^11^	*U*^22^	*U*^33^	*U*^12^	*U*^13^	*U*^23^
Br1	0.0454 (3)	0.0422 (3)	0.0301 (3)	0.0153 (2)	−0.0037 (2)	0.0094 (2)
N1	0.0305 (17)	0.0202 (15)	0.0147 (15)	0.0011 (16)	−0.0007 (14)	0.0015 (13)
N2	0.0227 (16)	0.045 (2)	0.0204 (16)	−0.0014 (16)	−0.0035 (14)	−0.001 (2)
C1	0.034 (2)	0.034 (2)	0.0178 (19)	0.001 (2)	−0.0023 (18)	−0.0059 (19)
C2	0.034 (2)	0.031 (2)	0.027 (2)	−0.008 (2)	0.0046 (17)	0.000 (2)
C3	0.035 (2)	0.0237 (18)	0.0174 (18)	0.0000 (17)	0.0033 (19)	−0.0012 (18)
C4	0.0308 (18)	0.0285 (19)	0.0122 (17)	0.0027 (17)	0.0021 (17)	−0.0017 (18)
C5	0.031 (2)	0.036 (2)	0.019 (2)	0.006 (2)	−0.0016 (16)	0.0021 (18)
C7	0.0233 (19)	0.030 (2)	0.0122 (17)	−0.0002 (18)	−0.0003 (16)	−0.0036 (17)
C8	0.033 (2)	0.033 (2)	0.0193 (19)	−0.009 (2)	0.0079 (17)	−0.0092 (19)
C9	0.039 (2)	0.026 (2)	0.0192 (19)	0.001 (2)	0.0065 (17)	−0.0019 (19)
C10	0.032 (2)	0.026 (2)	0.0149 (17)	0.0044 (18)	0.0018 (17)	0.0002 (17)
C11	0.0241 (19)	0.029 (2)	0.0146 (17)	−0.0013 (18)	0.0012 (16)	−0.0061 (17)
C12	0.0239 (17)	0.0251 (19)	0.0109 (16)	0.0044 (18)	0.0033 (14)	−0.0020 (17)
N3	0.0270 (17)	0.0253 (18)	0.0260 (18)	−0.0015 (16)	0.0049 (16)	0.0003 (16)
O1	0.0495 (18)	0.0319 (16)	0.057 (2)	0.0126 (15)	−0.0092 (19)	−0.0028 (18)
O2	0.0329 (15)	0.0293 (14)	0.0390 (17)	−0.0046 (13)	−0.0007 (13)	−0.0093 (17)
O3	0.0260 (14)	0.0357 (16)	0.0311 (15)	0.0003 (14)	−0.0033 (13)	−0.0038 (14)

### Geometric parameters (Å, °)

**Table:**

Br1—C10	1.896 (4)	C3—H3B	0.9700
N1—C1	1.491 (5)	C4—C5	1.375 (5)
N1—C2	1.492 (5)	C4—C12	1.437 (6)
N1—C3	1.521 (5)	C5—H5A	0.9300
N1—H1	0.9100	C7—C8	1.389 (6)
N2—C5	1.345 (6)	C7—C12	1.408 (5)
N2—C7	1.376 (5)	C8—C9	1.382 (6)
N2—H2D	0.8600	C8—H8A	0.9300
C1—H1A	0.9600	C9—C10	1.412 (6)
C1—H1B	0.9600	C9—H9A	0.9300
C1—H1C	0.9600	C10—C11	1.379 (6)
C2—H2A	0.9600	C11—C12	1.403 (5)
C2—H2B	0.9600	C11—H11A	0.9300
C2—H2C	0.9600	N3—O1	1.232 (4)
C3—C4	1.484 (5)	N3—O2	1.251 (4)
C3—H3A	0.9700	N3—O3	1.258 (4)
			
C1—N1—C2	110.6 (3)	C5—C4—C12	106.1 (3)
C1—N1—C3	112.8 (3)	C5—C4—C3	126.6 (4)
C2—N1—C3	110.5 (3)	C12—C4—C3	127.3 (3)
C1—N1—H1	107.6	N2—C5—C4	110.3 (4)
C2—N1—H1	107.6	N2—C5—H5A	124.9
C3—N1—H1	107.6	C4—C5—H5A	124.9
C5—N2—C7	109.7 (3)	N2—C7—C8	130.7 (4)
C5—N2—H2D	125.2	N2—C7—C12	107.2 (3)
C7—N2—H2D	125.2	C8—C7—C12	122.1 (4)
N1—C1—H1A	109.5	C9—C8—C7	118.3 (4)
N1—C1—H1B	109.5	C9—C8—H8A	120.8
H1A—C1—H1B	109.5	C7—C8—H8A	120.8
N1—C1—H1C	109.5	C8—C9—C10	119.4 (4)
H1A—C1—H1C	109.5	C8—C9—H9A	120.3
H1B—C1—H1C	109.5	C10—C9—H9A	120.3
N1—C2—H2A	109.5	C11—C10—C9	123.1 (4)
N1—C2—H2B	109.5	C11—C10—Br1	118.4 (3)
H2A—C2—H2B	109.5	C9—C10—Br1	118.5 (3)
N1—C2—H2C	109.5	C10—C11—C12	117.3 (4)
H2A—C2—H2C	109.5	C10—C11—H11A	121.4
H2B—C2—H2C	109.5	C12—C11—H11A	121.4
C4—C3—N1	113.2 (3)	C11—C12—C7	119.7 (4)
C4—C3—H3A	108.9	C11—C12—C4	133.5 (4)
N1—C3—H3A	108.9	C7—C12—C4	106.8 (3)
C4—C3—H3B	108.9	O1—N3—O2	121.3 (3)
N1—C3—H3B	108.9	O1—N3—O3	121.0 (3)
H3A—C3—H3B	107.8	O2—N3—O3	117.8 (3)
			
C1—N1—C3—C4	59.5 (4)	C8—C9—C10—Br1	179.3 (3)
C2—N1—C3—C4	−176.1 (3)	C9—C10—C11—C12	−0.5 (5)
N1—C3—C4—C5	−103.6 (4)	Br1—C10—C11—C12	179.8 (3)
N1—C3—C4—C12	78.1 (5)	C10—C11—C12—C7	2.0 (5)
C7—N2—C5—C4	0.0 (5)	C10—C11—C12—C4	−178.5 (4)
C12—C4—C5—N2	−0.3 (4)	N2—C7—C12—C11	179.2 (3)
C3—C4—C5—N2	−178.8 (4)	C8—C7—C12—C11	−2.7 (5)
C5—N2—C7—C8	−177.6 (4)	N2—C7—C12—C4	−0.5 (4)
C5—N2—C7—C12	0.3 (4)	C8—C7—C12—C4	177.7 (3)
N2—C7—C8—C9	179.4 (4)	C5—C4—C12—C11	−179.1 (4)
C12—C7—C8—C9	1.7 (6)	C3—C4—C12—C11	−0.6 (7)
C7—C8—C9—C10	−0.2 (6)	C5—C4—C12—C7	0.5 (4)
C8—C9—C10—C11	−0.4 (6)	C3—C4—C12—C7	179.0 (4)

### Hydrogen-bond geometry (Å, °)

**Table:**

*D*—H···*A*	*D*—H	H···*A*	*D*···*A*	*D*—H···*A*
N1—H1···O2	0.91	2.24	3.041 (4)	146
N1—H1···O3	0.91	2.03	2.857 (4)	151
N1—H1···N3	0.91	2.49	3.391 (5)	169
N2—H2D···O2^i^	0.86	2.12	2.902 (4)	152
N2—H2D···O1^i^	0.86	2.65	3.388 (4)	144
C1—H1B···O3^ii^	0.96	2.45	3.293 (5)	146
C3—H3B···O3^ii^	0.97	2.40	3.259 (5)	147

Symmetry codes: (i) *x*−1, *y*, *z*; (ii) −*x*, *y*+1/2, −*z*+1/2.
